# The Simultaneous Voltammetric Determination of Aflatoxins В_1_ and М_1_ on a Glassy-Carbon Electrode

**DOI:** 10.1155/2018/6285623

**Published:** 2018-05-09

**Authors:** G. B. Slepchenko, T. M. Gindullina, M. A. Gavrilova, A. Zh. Auelbekova

**Affiliations:** National Research Tomsk Polytechnic University, Tomsk 634050, Russia

## Abstract

For the first time, the possibility of using stripping voltammetry for the simultaneous determination of aflatoxins В_1_ and М_1_ on a glassy-carbon electrode has been shown. The influence of various factors (*Е*_э_, *τ*_э_, *w*, and the nature of the background electrolyte) on the potential and magnitude of the oxidation current of mycotoxins has been estimated. Working conditions for voltammetric determination and reproducibility of analytical signals for two mycotoxins have been selected. The mutual influence of aflatoxins В_1_ and М_1_ on the value of analytical signals in their simultaneous presence has been studied. It has been found that, in the range of their detectable contents, the presence of aflatoxin В_1_ reduces the analytical signal of aflatoxin М_1_ by 45–50%, but the linearity of the calibration dependence is preserved. The content of aflatoxin М_1_ in determination of aflatoxin B_1_ does not exert a significant effect in the range of 10–15%. Based on the results obtained, a procedure has been proposed for determining the content of aflatoxins В_1_ and М_1_ in their joint presence in milk by voltammetry in the concentration ranges 2 × 10^−3^ ÷ 2 × 10^−1^ mg/dm^3^ and 2 × 10^−4^ ÷ 2 × 10^−2^ mg/dm^3^, respectively (Sr not more than 18%).

## 1. Introduction

**Figure 1:**
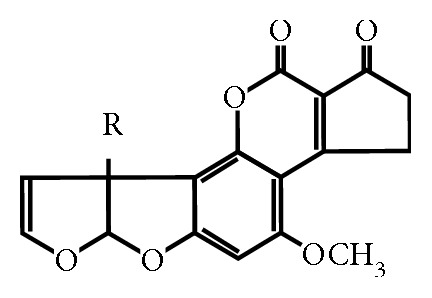
Structural formula of aflatoxins В_1_ (R=H) and М_1_ (R=ОН).

Aflatoxin M_1_ is a metabolite of aflatoxin B_1_, a product of life activity of *Aspergillus* microscopic fungi. In natural conditions, aflatoxin B_1_ contaminates cereals, legumes, various nuts, oil seeds, cocoa and coffee, animal feed, and other food products. It can be converted into aflatoxin M_1_ in the body of animals and is present in meat [1]. In [2], the information on admissible levels of contents of the specified kinds of mycotoxins in flour obtained from various kinds of grains used for cooking human food, as well as in the composition of feed of various animal species, is presented. With contaminated feed, aflatoxins enter the body of animals, and their residual quantities are found in meat, milk, and eggs. Aflatoxins are the only mycotoxins that are strictly regulated in markets such as the EU and US [3]. The information on tolerable levels of mycotoxins taken in Australia, China, Guatemala, India, Ireland, Kenya, and Taiwan is reported [4]. Aflatoxin M_1_ is a hydroxylated metabolite present in human milk and animals exposed to aflatoxin B_1_. Like its precursor, aflatoxin B_1_, aflatoxin M_1_ already in low concentrations poses a serious threat to the health of animals and humans. Aflatoxin B_1_ is found not only in whole milk (including reconstituted milk) but also in cottage cheese, cheese, and yoghurt. Dairy products contaminated with aflatoxin M_1_ are environmentally hazardous to humans. In adult food and baby food, aflatoxins are not allowed.  Figure 1 shows the structural formula of aflatoxins B_1_ and M_1_.

Aflatoxin B_1_ is (6aR-*cis*)(2,3,6a,9a)-tetrahydro-4-methoxycyclopenta[c]furo[2,3-h][1]benzopyran-1,11-dione with a molecular weight of 312. Aflatoxin M_1_ is (2,3,6a,9a)-tetrahydro-9a-hydroxy-4-methoxycyclopenta[c]furo[2,3-h][1]benzopyran-1,11-dione with a molecular weight of 328.

There are known methods of simultaneous quantitative determination of aflatoxins B_1_ and M_1_, aflatoxins B_1_, В_2_, G_1_, G_2_, and M_1_, and ochratoxin A by the method of high-performance liquid chromatography with fluorescent detection in breast milk [5–8] with detection limits between 0.5 and 0.25 *μ*g/L [7], of 5 ng/L [7], and from 0.005 to 0.03 ng/mL [8].

The method of high-performance liquid chromatography was used to determine aflatoxin M_1_ in eggs [9], and together with tandem mass-spectrometry, it was used to determine aflatoxins B_1_ and M_1_ in fresh and dry milk after ultrasonic extraction; the detection limit was 0.05 *μ*g/kg, and the limit of quantification was 0.1 *μ*g/kg [10]. The detection limits of aflatoxins B_1_, В_2_, G_1_, G_2_, and M_1_ and ochratoxin A in food products of animal origin were in the range of 0.07–0.59 *μ*g/kg [11]. In case of simultaneous determination of six aflatoxins (B_1_, В_2_, G_1_, G_2_, M_1,_ and M_2_) by this method in peanut, the detection limits are in the range of 0.03 to 0.26 ng/g and 0.1 to 0.88 ng/g[12]. The use of chromatography methods is complicated by the duration and the need to use expensive equipment and highly toxic solvents as a mobile phase.

The possibility of indirect competitive enzyme-linked immunosorbent assay for the determination of aflatoxin M_1_ in various objects was demonstrated in [13]. At present, highly sensitive, inexpensive, and easy-to-use electrochemical methods, in particular voltammetry, are becoming increasingly popular for the determination of a number of organic substances including aflatoxins. In literature, there is a rather large number of works on the individual determination of aflatoxins B_1_ [14–19] and M_1_ [20–23] using amperometric and voltammetric immunosensors. The use of enzymes and nanomaterials to design sensors provides high sensitivity and selectivity for detection. At the same time, the analysis of numerous publications in the databases of Science Direct, Scopus, Web of Science, and so on shows that, at the moment, there is no research work on the simultaneous quantification of aflatoxins В_1_ and М_1_ by the method of voltammetry.

*The purpose of the work* consists of studying the possibility of simultaneous voltammetric determination of aflatoxins В_1_ and М_1_ on a glassy-carbon electrode (GCE), selecting working conditions for measurements and developing a method for their determination in whole milk.

## 2. Methods and Materials

In this study, the voltammetric analyzer “STA” (Russia) consisting of electronic and measuring units and an IBM-compatible personal computer with the installed software package “STA” was used. As the indicator electrode, a glassy-carbon electrode (GCE) was used, and the conventional silver chloride electrode (CSE) served as an auxiliary and reference electrode.

The measurements were carried out in a constant-current sweep mode with the speed *w* = 30 mV/s in the potential range from 0.0 to +1.1 V. To mix the analyzed solution, vibration of the electrodes without removal of dissolved oxygen was used.

The working solutions of aflatoxins В_1_ and М_1_ were prepared from standard samples of aflatoxin В_1_ (GSO 7936-2001) with a concentration of 10.0 mg/dm^3^ and aflatoxin М_1_ (GSO 7935-2001) with a concentration of 1.0 mg/dm^3^ in the volume of 1.0 dm^3^ mixture of benzene and acetonitrile (in the 9 : 1 ratio), followed by diluting 10 times in ethyl alcohol. As background electrolytes, solutions with different pH: 0.1 M Na_2_HPO_4_, 0.1 М С_6_Н_5_О_7_(NH_4_)_3_, 0.1 М (NH_4_)_2_SO_4_, 0.1 M Na_3_PO_4_, 0.1 M K_2_HPO_4_, 0.1 M Li_2_CO_3_, and 0.1 М ZnSO_4_, were used.

## 3. Preparation of the Sample of Whole Milk

When preparing for analysis, a sample of whole milk 25.00 g is taken in a conical flask with the capacity of 100 cm^3^, and 1.0 cm^3^ of hydrochloric acid with a concentration of 6–7 mol/dm^3^ is added in portions of 0.2 cm^3^. The mixture is slightly stirred and left for 15 minutes, poured into centrifuge tubes, and then centrifuged at 15,000 rpm within 15 minutes.

The centrifugate is poured into a conical flask, and 5-6 g of ammonium sulfate ((NH)_2_SO_4_) is added in portions of 2 to 3 grams, each time stirring the contents of the flask with a glass rod until the salt dissolves. The flask is left for 20 minutes, after which the contents of the flask are poured into centrifuge tubes and centrifuged within 15 minutes at the speed of 6000 rpm. The centrifugate is filtered into a clean cup with the capacity of 30 cm^3^ through the double-layered filter paper (blue tape). The resulting filtrate is a prepared sample. For analysis, an aliquot of the prepared sample of 5.0 cm^3^ is taken.

## 4. Results and Discussion

**Figure 2:**
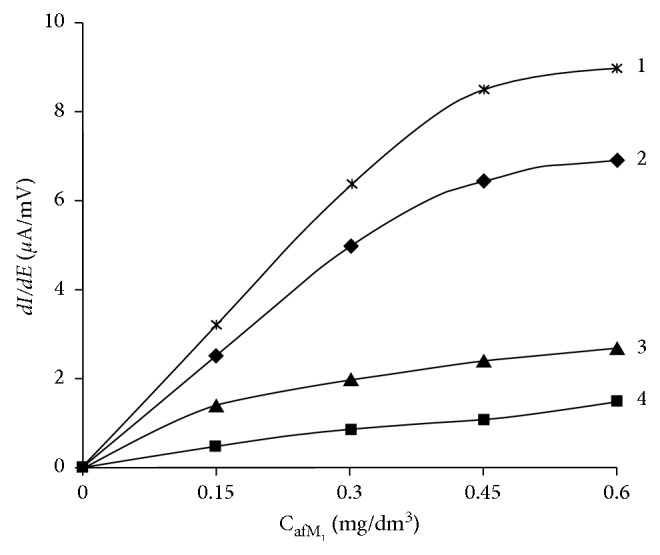
Calibration dependences of electric oxidation of aflatoxins М_1_ (1 and 3) and В_1_ (2 and 4) on various background electrolytes: (1, 2) 0.1 М С_6_Н_5_О_7_(NH_4_)_3_; (3, 4) 0.1 М (NH_4_)_2_SO_4_.

Studies on the effect of the background electrolyte composition on the analytical signals of aflatoxins В_1_ and М_1_ on a glassy-carbon electrode under working conditions previously developed for the determination of aflatoxin В_1_ were conducted [24]. Experiments on the choice of the background electrolyte showed that the value of the analytical signal aflatoxin M_1_ on background electrolytes: 0.1 M Na_3_PO_4_, 0.1 M Na_2_HPO_4_, 0.1 M K_2_HPO_4_, and 0.1 M ZnSO_4_, was found to be low, and it was high on background electrolytes: 0.1 M (NH_4_)_2_SO_4_ and 0.1 M Li_2_CO_3_; the maximum current of its electric oxidation was obtained against the background of 0.1 M C_6_H_5_O_7_(NH_4_)_3_. Changing the cation-anion composition and pH of the background electrolyte may negatively shift the peak potential of the electric oxidation peak of aflatoxin М_1_ in the range (0.6 ± 0.08) V. The effect of the background electrolyte pH on the analytical signals of these aflatoxins was studied, and it was shown that it was preferable to use neutral or weak acidic solutions as background ones, since mycotoxins decompose into nontoxic or low-toxic compounds in the alkaline medium, and the use of background electrolytes with pH > 6.5 is impractical. In Figure 2, calibration curves of the peak current of electric oxidation of aflatoxins В_1_ and М_1_ in various background electrolytes are presented. According to the calibration curves, two background electrolytes were selected: 0.1 M 3-substituted ammonium citrate and 0.1 M ammonium sulfate solution, providing a high detection sensitivity coefficient in the range of determined contents 2 × 10^−4^ ÷ 0.6 mg/dm^3^.

**Figure 3:**
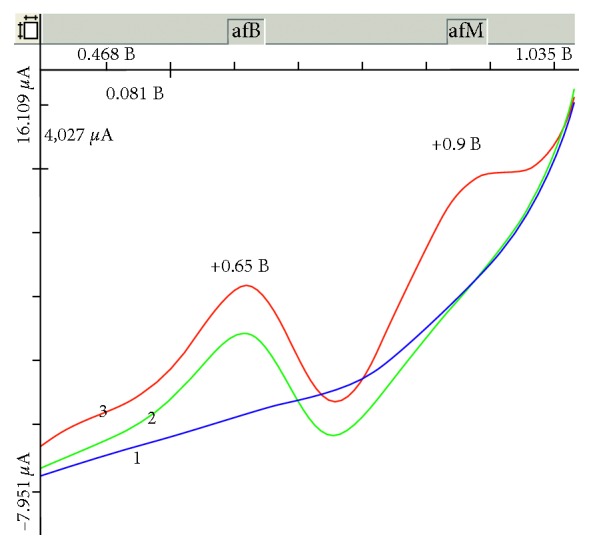
Voltammograms of aflatoxins В_1_ and М_1_ electric oxidation with the joint presence on the GCE: (1) background electrolyte 0.1 М С_6_Н_5_О_7_(NH_4_)_3_; (2) *C*_afB_1__ = 2 × 10^−3^ mg/dm^3^ and *C*_afM_1__ = 0; (3) *C*_afB_1__ = 2 × 10^−3^ mg/dm^3^ and *C*_afM_1__ = 2 × 10^−4^ mg/dm^3^.

In Figure 3, voltammograms of the electric oxidation of aflatoxins В_1_ and М_1_ on the GCE in the selected background electrolyte are shown. Analytical signals are well separated and reproduced.

Both electrolytes can be used for the joint quantification of mycotoxins, but 0.1 М С_6_Н_5_О_7_(NH_4_)_3_ was selected as the working background electrolyte providing sufficient resolution and satisfactory reproducibility of the analytical signal.

**Figure 4:**
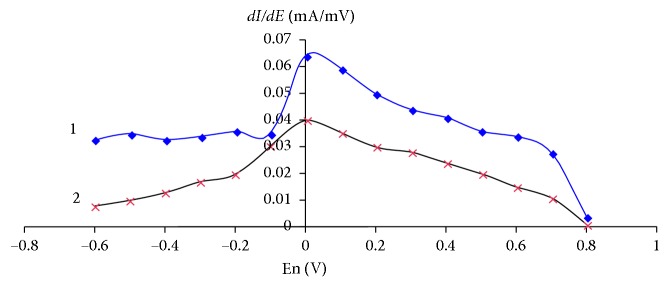
Dependences of the current derivatives of the aflatoxins В_1_ and М_1_ peak on the accumulation potential of the GCE. The background electrolyte is 0.1 М С_6_Н_5_О_7_(NH_4_)_3_; *τ*_э_ = 30 s; *w* = 30 mV/s; (1) *C*_afM_1__ = 2 × 10^−3^ mg/dm^3^; (2) *C*_afM_1__ = 2 × 10^−4^ mg/dm^3^.

Figure 4 shows the dependences of the current derivatives of the peak of aflatoxins В_1_ (curve 1) and М_1_ (curve 2) on the accumulation potential of the GCE in the selected background electrolyte. It can be seen in Figure 4 that the maximum values of the electric oxidation currents of aflatoxins are observed at the potential of 0.0 V that was selected as the accumulation potential for further studies.

**Figure 5:**
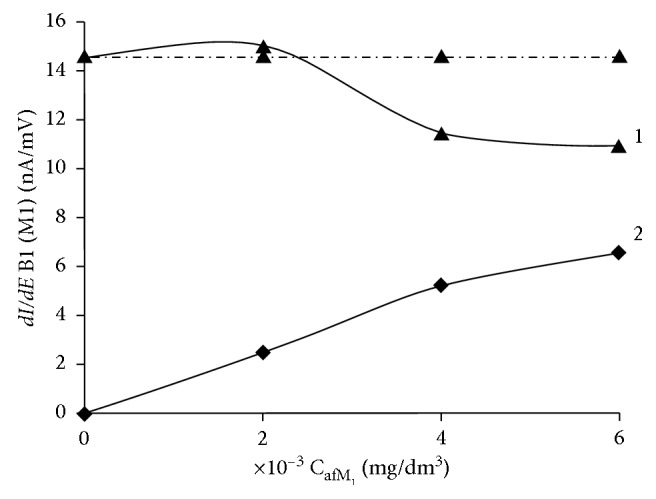
Dependences of the current derivatives of the peak of aflatoxins В_1_ (1) and М_1_ (2) on the aflatoxin М_1_ content in the background electrolyte on the GCE: the background electrolyte is 0.1 М С_6_Н_5_О_7_(NH_4_)_3_; (1) *C*_afB_1__ = 2 × 10^−3^ mg/dm^3^ and *C*_afM_1__ = var (on 2 × 10^−3^ mg/dm^3^); (2) dependence of *I*_p·afM_1__ on *C*_afM_1__.

**Figure 6:**
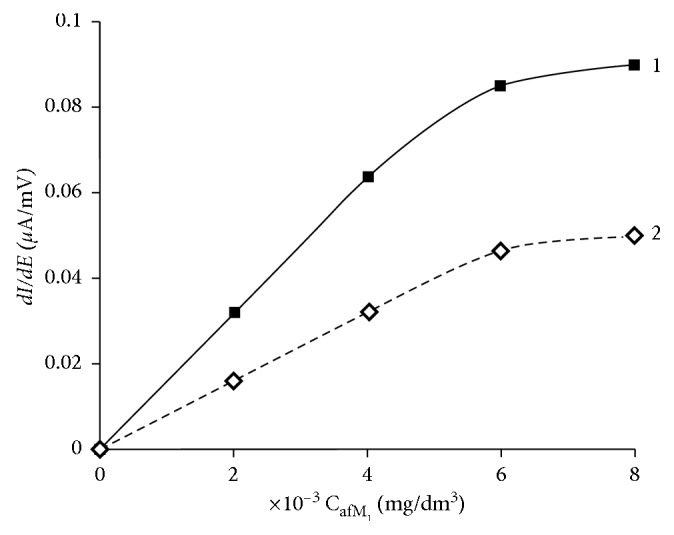
Calibration dependences of aflatoxin М_1_ on the GCE: the background electrolyte is 0.1 М С_6_Н_5_О_7_(NH_4_)_3_; *Е*_е_ = 0,0 В; *τ*_е_ = 30 s; (1) *C*_afB_1__ = 0; (2) *C*_afB_1__ = 2 × 10^−3^ mg/dm^3^.

The mutual influence of aflatoxins В_1_ and М_1_ in their simultaneous determination on a glassy-carbon electrode was studied. For this purpose, the current derivative of the peak of aflatoxin В_1_ electric oxidation was obtained as a function of the concentration of aflatoxin М_1_ in the solution (Figure 5) and the calibration curves of aflatoxin М_1_ in the presence of aflatoxin В_1_ (Figure 6).

In Figure 5, it can be seen that, in the concentration range studied, the effect of aflatoxin M_1_ on the aflatoxin B_1_ current is practically negligible at the ratio *C*_aΦB_1__ : *C*_aΦM_1__=1 : 1 in the presence of a two- or threefold excess of aflatoxin M_1_, the peak current of aflatoxin B_1_ decreases by 10–15% (curve 1), and the potential of the peak remains unchanged. It is shown that the systematic error in determination of aflatoxin B_1_ in the presence of aflatoxin M_1_ at the ratio *C*_B_1__/*C*_M_1__ ≤ 1 : 40 does not exceed 20%.

In Figure 6, it is seen that, in the presence of aflatoxin B_1_, the derivative of the peak current of aflatoxin M_1_ decreases almost 1.5 times and the peak potential shifts to the anode region from +0.85 V to +0.95 V, but the linearity of the calibration dependence remains in a wide range which proves the possibility of their simultaneous determination.

Based on the conducted studies, the working conditions of the simultaneous voltammetric determination of aflatoxins B_1_ and М_1_ were proposed (Table 1).

On the basis of the obtained data of the electrochemical behavior of aflatoxins, an algorithm for quantifying these toxic substances in order to effectively control the detection of their minimum acceptable amounts in whole milk was developed. The algorithm for quantification of mycotoxins in whole milk includes the following steps:

1. Taking a sample
2. Acid hydrolysis with concentrated HCl and centrifugation
3. Precipitation of proteins with ammonium salts of sulfate (NH_4_)_2_SO_4_ and centrifugation
4. Filtration of the obtained precipitate
5. Quantitative determination of the aflatoxins content by the method of differential voltammetry

Verification of the correctness of the proposed procedure was carried out by the “introduced-found” method (Table 2).

The data in Table 2 show that the voltammetric joint for determination of quantities of aflatoxins B_1_ and M_1_ is possible with the measurement error of 15–20% in the concentration ranges 2 × 10^−3^ ÷ 2 × 10^−1^ mg/dm^3^ and 2 × 10^−4^ ÷ 2 × 10^−2^ mg/dm^3^, respectively.

The proposed method is simple, and it does not require a lot of reagents and labor. The range of detectable concentrations is from 0.001 to 0.12 mg/dm^3^. The relative standard deviation (Sr) is not more than 30%.

## 5. Conclusion

Thus, the possibility of the simultaneous voltammetric determination of aflatoxins B_1_ and M_1_ on the GCE in the background electrolyte 0.1 М С_6_Н_5_О_7_(NH_4_)_3_ has been shown.

When determining aflatoxins, the method of “soft” sample preparation has been used for separating the matrix by hydrolysis and salting out proteins followed by their separation by centrifugation or filtration which reduces the analysis time to less than one hour as compared with thin-layer and high-performance chromatography (GOST 30711-2001). The developed technique has a number of advantages in comparison with the already known methods of analysis. The algorithm of the technique is characterized by the express analysis (the analysis time does not exceed 1 hour), sensitivity (the range of the determined contents is not inferior, and in the case of aflatoxin M_1_, it exceeds the capabilities of chromatographic methods), and the equipment cheapness. The technique is characterized by simplicity of execution, minimal consumption of reagents, and improved metrological characteristics.

## Figures and Tables

**Table 1:** Working conditions of the simultaneous voltammetric determination of aflatoxins В_1_ and М_1_.

Parameters of votammetric determination of aflatoxins	Parameters values	
	В_1_	М_1_
The system used	3-electrode	
Electrodes		
(i) Indicator	GCE	
(ii) Comparison/auxiliary	CSE/CSE	
Background electrolyte	0.1 М С_6_Н_5_О_7_(NH_4_)_3_	
Electrolysis potential *Е*_э_ (V)	0,0	
Potential sweeping range (V)	0,0 ÷ +1,1	
Potential changing speed *w* (mV/s)	30	
Registration mode	Differential	
Peak potential *Е*_п_ (V)	+0.65 ± 0.05	+0.90 ± 0.05

**Table 2:** Verification of correctness of the voltammetric method for determination of the content of aflatoxins B_1_ and M_1_ in model samples of whole milk by the “introduced-found” method (*P*=0.95and*n*=5).

Object	Component	Content of aflatoxins В_1_(10^3^) and М_1_(10^4^) (mg/dm^3^)		
		In samples	Introduced	Found
Cow's milk	В_1_	2.79 ± 0.41	2.00	4.81 ± 0.72
Fat content: 3.8%	М_1_	3.01 ± 0.45	2.00	5.43 ± 0.81
Cow's milk	В_1_	1.94 ± 0.35	2.00	3.95 ± 0.65
Fat content: 1.5%	М_1_	2.12 ± 0.32	2.00	4.21 ± 0.98
Goat's milk	В_1_	3.92 ± 0.58	4.00	7.85 ± 1.12
Fat content: 4.4%	М_1_	2.08 ± 0.31	4.00	5.95 ± 0.91
Sour milk	В_1_	13.5 ± 1.9	10.0	22.6 ± 3.1
Fat content: 2.5%	М_1_	15.3 ± 2.1	10.0	26.1 ± 3.7

## References

[1] Codex Stan 193-1995 (1995).

[2] Wu F. (2004). Mycotoxin risk assessment for the purpose of setting international regulatory standards.

[3] The Commission of the European (2006).

[4] Coulter J. B., Lamplugh S. M., Suliman G. I., Omer M. I., Hendrickse R. G. (1984). Aflatoxins in human breast milk.

[5] Scaglioni P. T, Becker-Algeri T., Drunkler D., Badiale-Furlong E. (2014). Aflatoxin B1 and M1 in milk.

[6] Adejumo O., Atanda O., Raiola A., Somorin Y., Bandyopadhyay R., Ritieni A. (2013). Correlation between aflatoxin M1 content of breast milk, dietary exposure to aflatoxin B1 and socioeconomic status of lactating mothers in Ogun State, Nigeria.

[7] Gürbay A., Sabuncuoğlu S. A., Girgin G. (2010). Exposure of newborns to aflatoxin M1 and B1 from mothers’ breast milk in Ankara, Turkey.

[8] Andrade P. D., Gomes da Silva J. L., Caldas E. D. (2013). Simultaneous analysis of aflatoxins B1, B2, G1, G2, M1 and ochratoxin A in breast milk by high-performance liquid chromatography/fluorescence after liquid–liquid extraction with low temperature purification (LLE–LTP).

[9] Wacoo A. P., Wendiro D., Vuzi P. C., Hawumba J. F. (2014). Methods for detection of aflatoxins in agricultural food crops.

[10] Fan S., Li Q., Sun L., Du Y., Xia J., Zhang Y. (2015). Simultaneous determination of aflatoxin B1and M1 in milk, fresh milk and milk powder by LC-MS/MS utilising online turbulent flow chromatography.

[11] Dongmei C., Xiaoqin C., Yanfei T. (2012). Development of a sensitive and robust liquid chromatography coupled with tandem mass spectrometry and a pressurized liquid extraction for the determination of aflatoxins and ochratoxin A in animal derived foods.

[12] Sartori A. V., de Mattos J. S., Souza Y. P., Pereira dos Santos R., de Moraes M. H. P., da Nóbrega A. W. (2015). Determination of aflatoxins M1, M2, B1, B2, G1 and G2 in peanut by modified QuEChERS method and ultra-high performance liquid chromatography-tandem mass spectrometry.

[13] Radoi A., Targa M., Prieto-Simon B., Marty J.-L. (2008). Enzyme-linked immunosorbent assay (ELISA) based on superparamagnetic nanoparticles for aflatoxin M1 detection.

[14] Linting Z., Ruiyi L., Zaijun L., Qianfang X., Yinjun F., Junkang L. (2012). An immunosensor for ultrasensitive detection of aflatoxin B1 with an enhanced electrochemical performance based on graphene/conducting polymer/gold nanoparticles/the ionic liquid composite film on modified gold electrode with electrodeposition.

[15] Masoomi L., Sadeghi O., Banitaba M. H., Shahrjerdi A., Davarani S. S. H. (2013). A non-enzymatic nanomagnetic electro-immunosensor for determination of aflatoxin B1 as a model antigen.

[16] Maa H., Suna J., Zhanga Y., Biana C., Xiaa S., Zhen T. (2016). Label-free immunosensor based on one-step electrodeposition of chitosan-gold nanoparticles biocompatible film on Au microelectrode for the determination of aflatoxin B1 in maize.

[17] Sharma A., Kumar A., Khan R. (2017). Electrochemical immunosensor based on poly(3,4-ethylenedioxythiophene) modified with gold nanoparticle to detect aflatoxin B1.

[18] Abnous K., Danesh N. M., Alibolandi M. (2017). A new amplified π-shape electrochemical aptasensor for ultrasensitive detection of aflatoxin B1.

[19] Mavrikoua S., Flampouria E., Iconomoub D., Kintziosa S. (2017). Development of a cellular biosensor for the detection of aflatoxin B1, based on the interaction of membrane engineered Vero cells with anti-AFB1 antibodies on the surface of gold nanoparticle screen printed electrodes.

[20] Paniel N., Radoi A., Marty J.-L. (2010). Development of an electrochemical biosensor for the detection of aflatoxin M1 in milk.

[21] Parker C. O., Lanyon Y. H., Manning M., Arrigan D. W., Tothill I. E. (2009). Electrochemical immunochip sensor for aflatoxin M1 detection.

[22] Parker C. O., Tothill I. E. (2009). Development of an electrochemical immunosensor for aflatoxin M1 in milk with focus on matrix interference.

[23] Istambouliéa G., Paniela N., Zaraa L. (2016). Development of an impedimetric aptasensor for the determination of aflatoxin M1 in milk.

[24] Gavrilova M. A., Slepchenko G. B., Mikheeva E. V., Deryabina V. I. (2014). Voltammetric determination of aflatoxin B1.

